# Unaccounted immunosuppression and absent donor data limit infection risk after high-urgency heart transplantation

**DOI:** 10.1093/eschf/xvag182

**Published:** 2026-06-24

**Authors:** Shankar Biswas, Harshitha Tallapally, Yashasvi Srivastava, Saikiran Budige

**Affiliations:** Department of Internal Medicine, Ivano-Frankivsk National Medical University, Halytska Street, 2, Ivano-Frankivsk 76000, Ukraine; Internal Medicine, Osmania Medical College, Hyderabad, Telangana, India; Internal Medicine, Ivano-Frankivsk National Medical University, Ivano-Frankivsk 76000, Ukraine; Internal Medicine, Kamineni Institute of Medical Sciences, Hyderabad, Telangana, India

**Keywords:** Heart transplantation, Immunosuppression, Post-transplant infection, Corticosteroids, Donor characteristics, Mechanical circulatory support

We read with interest the work by Lee KS *et al*.^1^ evaluating outcomes in 801 Korean Organ Transplant Registry recipients after transplantation stratified by KONOS urgency status. One-year mortality was significantly greater among Status 0 patients [28.0% vs 13.5%; HR 2.26 (95% CI 1.57–3.25), *P* < .001] and infection post-transplant accounted for 47.4%, 34.9%, and 34.2% of this excess at 1, 6, and 12 months, respectively. We have two methodological issues with the multivariable infection models that are relevant to the scope of the primary conclusion of the study.

The authors note that when considering pre-transplant mechanical circulatory support and extended post-operative mechanical ventilation (>24 h), urgency status no longer predicted 1-month infection [HR 1.15 (95% CI 0.58–2.27), *P* = .680], suggesting that mechanical support burden and ventilator duration, rather than urgency status *per se*, explains the excess 1-month infection. This may be true, but the multivariable infection models in Supplementary Table S4 failed to include immunosuppression intensity as a time-varying covariate despite demonstrating consistent and significantly different patterns of immunosuppression in the two groups themselves.

The data in Table 3 indicates that Status 0 patients had significantly higher corticosteroid doses at both 6 months (8.2 ± 10.1 vs 6.0 ± 3.6 mg; *P* = .001) and 1 year post-transplant (6.1 ± 7.0 vs 4.1 ± 1.7 mg, *P* < .001) and received lower doses of tacrolimus and lower doses of mycophenolate during the same period. The difference in corticosteroid dose was not an artefact of discharge data but persisted throughout the first year post-transplant. Supplementary Figure S9A reveals a significant interaction term (*P* for interaction = 0.008) where failure to taper corticosteroids to ≤5 mg/day resulted in a higher risk of 6-month infection specifically in Status 0 patients. Post-heart transplant corticosteroid dose has been independently associated with the composite outcome, which includes post-transplant infection, in Cox multivariable models^2^ and post-transplant infection is also known to occur when recipients are over-immunosuppressed.^3^ Both of these metrics are available within the KOTRY cohort but were not incorporated as time-varying covariates in Supplementary Table S4, suggesting incomplete adjustment.

A second structural concern regards the absence of any donor characteristic from the manuscript and from entry into any multivariate models: neither donor age, cause of death, ischaemic time nor donor infectious history is present and no mention is made of how such variables were accounted for. Consequently, this analysis does not provide information regarding the independent effect of donor quality on increased infection among Status 0 patients; while the authors mention residual confounding in their discussion, this variable is not a remainder, but a fundamental gap which a multivariate approach cannot address.

We acknowledge the limitations of this type of response. The study utilized an established database and we have not been provided with the raw data. However, the interaction between time-varying corticosteroid tapering and urgency is itself reported by the authors; this variable should have been tested in the primary multivariate infection models and failure to incorporate it into the analyses represents an opportunity to test a key interaction within this cohort. Inclusion of donor-specific information in multivariable Cox analyses, either as time-varying immunosuppression or donor quality, may more effectively address questions of confounding if this data exists in any portion of the KOTRY database.

The study is valuable in characterizing urgency-stratified outcomes. We caution interpretation of the authors’ conclusion that mechanical support, rather than urgency status *per se*, explains infection post-transplant, as these analyses lack control for time-varying immunosuppression and donor quality data.

## Declarations

### Data Availability

No data were generated or analysed for this manuscript.
